# Single nucleotide polymorphisms associated with postoperative inadequate analgesia after single-port VATS in Chinese population

**DOI:** 10.1186/s12871-020-0949-6

**Published:** 2020-02-05

**Authors:** Xiufang Xing, Yongyu Bai, Kai Sun, Min Yan

**Affiliations:** Department of Anesthesiology and Pain Medicine, Second Affiliated Hospital, Zhejiang University School of Medicine, No.88 Jiefang Road, Hangzhou, 310009 China

**Keywords:** Postoperative pain, Single nucleotide polymorphism, Single-port video-assisted thoracoscopic surgery

## Abstract

**Background:**

Postoperative inadequate analgesia following video-assisted thoracoscopic surgery (VATS) is a common and significant clinical problem. While genetic polymorphisms may play role in the variability of postoperative analgesia effect, few studies have evaluated the associations between genetic mutations and inadequate analgesia after single-port VATS.

**Methods:**

Twenty-eight single nucleotide polymorphisms (SNPs) among 18 selected genes involved in pain perception and modulation were genotyped in 198 Chinese patients undergoing single-port VATS. The primary outcome was the occurrence of inadequate analgesia in the first night and morning after surgery which was defined by a comprehensive postoperative evaluation. Multivariable logistic regression analyses were used to identify the association between genetic variations and postoperative inadequate analgesia.

**Results:**

The prevalence of postoperative inadequate analgesia was 45.5% in the present study. After controlling for age and education level, association with inadequate analgesia was observed in four SNPs among three genes encoding voltage-gated sodium channels. Patients with the minor allele of rs33985936 (*SCN11A*), rs6795970 (*SCN10A*), and 3312G > T (*SCN9A*) have an increased risk of suffering from inadequate analgesia. While the patients carrying the minor allele of rs11709492 (*SCN11A*) have lower risk experiencing inadequate analgesia.

**Conclusions:**

We identified that SNPs in *SCN9A*, *SCN10A*, and *SCN11A* play a role in the postoperative inadequate analgesia after single-port VATS. Although future larger and long-term follow up studies are warranted to confirm our findings, the results of the current study may be utilized as predictors for forecasting postoperative analgesic effect for patients receiving this type of surgery.

**Trial registration:**

This study was retrospectively registered in the ClinicalTrials.gov Registry (NCT03916120) on April 16, 2019.

## Background

Thoracotomy is considered to be one of the most painful of surgical procedures [1]. Even though video-assisted thoracoscopic surgery (VATS) is less invasive and is generally expected to induce lower pain intensity, the moderate to severe postoperative pain remains common after VATS [2, 3]. The postoperative pain not only causes respiratory complications but negatively affects long-term rehabilitation [4, 5].

Multiple factors have been reported to affect pain sensitivity after surgery, such as age, gender, ethnicity, and type of surgery [6]. Recent advances in genetic research have shown that genetic polymorphisms may also play a role in the variability of pain perception [7–9]. Opioid receptor mu 1 (*OPRM1*) encodes the mu opioid receptor in humans, and plays an important role in endogenous pain modulation and opioid analgesia. Four single nucleotide polymorphisms (SNPs) in *OPRM1* were found significantly associated with higher pain intensity after thoracotomy [8]. Zhonghai Zhao et al. indicated that patients with mutant homozygous rs2032582 and rs1128503 loci in the *ABCB1* gene consumed more sufentanil at 6 h, 24 h and 48 h after thoracoscopic-assisted radical resection [9]. Besides, patients with the UGT2B7*2/*2 genotype had a higher risk of suffering severe pain 48 h after surgery [10]. Jin Ma et al. found that rs1718125 polymorphism in *P2RX7* gene had significant association with postoperative pain intensity and the consumption of fentanyl in patients undergoing lung resection [11].

The mechanism of acute pain after thoracotomy has not been fully illuminated, but it is believed to be caused by a variety of factors including the local damage of rib and skin incision, the inflammation caused by injury, and the acute intercostal neuralgia [1, 12]. The multifactorial nature of postoperative pain suggests that a number of distinct genetic factors may contribute to the variability in pain perception and analgesic effect after thoracotomy. Although more and more genetic polymorphisms have been identified as risk factors for rare and common pain syndromes [13, 14], most of these genes have not been studied in thoracotomy subjects.

In the present study, except for *OPRM1, ABCB1, UGT2B7, and P2RX7*, we selected other 14 genes known to be involved in systems related to pain perception and modulation based on evidence in the literature. The selected genes have been related to the ion channels (*SCN9A*, *SCN10A*, *SCN11A*, *KCNJ6*, *TRPV1*, and *CACNA1E*) [15–20], dopaminergic system (*COMT*, *DRD2*) [21, 22], purinergic receptor (*P2RY12*) [23], adrenergic receptor (*ADRB1*) [24], estrogen receptor (*ESR1*) [25], serine/threonine kinase (*TAOK3*) [26], growth factors (*TGFB1*) [27], and transcription factor (*CREB1*) [28]. The aim of the present study was to evaluate the association of common SNPs among aforementioned genes with the inadequate analgesia after single-port VATS.

## Methods

The current prospective study was approved by the Ethics Committee of the Second Affiliated Hospital of Zhejiang University School of Medicine, Hangzhou, Zhejiang, China, and the protocol was registered in the ClinicalTrials.gov Registry (NCT03916120). All subjects signed informed consent documents prior to enrollment.

### Patient characteristics

Two hundred Thirty-two subjects were recruited from consecutive patients undergoing selective lung section with single-port VATS performed by one attending surgeon at the Second Affiliated Hospital of Zhejiang University School of Medicine between July 2018 and January 2019. The detailed surgical procedure was previously described [29]. The criteria for inclusion in the study were age from 18 to 70, ASA classification I to III, and voluntarily received patient-controlled intravenous (PCIA) treatment. The exclusion criteria included the following: (1) history of mental illness, chronic pain, and alcohol or drug abuse; (2) remarkably abnormal liver and/or kidney function (more than two times of the normal); (3) allergy to related opioid drugs; (4) women during pregnancy or lactation.

### Anesthesia protocol

All patients received general anesthesia under standard protocol. Specifically, general anesthesia was induced with midazolam (0.2 mg*kg-1), sufentanil (10 μg*kg-1), and etomidate (0.3 mg*kg-1). Cisatracurium besilate (0.15 mg*kg-1) was administered to induce a neuromuscular blockade for tracheal intubation. Anesthesia was continuously maintained with sevoflurane, propofol, and remifentanil. Cisatracurium was bloused as needed. During the surgery, standardized monitoring and bispectral index were applied. Central venous catheterization (CVC) and A-line were implemented for each patient. Before closure of the thoracic incision, surgeons performed a three-site intercostal nerve block with 0.75% 10 mL ropivacaine under thoracoscope. At the end of surgery, pentazocine 5 mg and tropisetron 5 mg were administered by the anesthetist. Immediately after surgery, PCIA was connected to the CVC. Then, patients were transferred to postanesthesia care unit (PACU) for recovery where their vital signs were continuously monitored.

### Postoperative pain management

Each subject was extubated at PACU when vital signs stabilized. Patients were asked every 10–15 min after they were awake enough whether they needed pain medication until they became conscious enough to use the PCIA. If the patients felt moderate or severe pain (visual analog scale [VAS] 40–100, 0 = no pain to 100 = intense pain), they were given 40 mg dynastat until their VAS was ≤30. Patients were excluded if they received dynastat as rescue analgesia at PACU. PCIA was administered with a bolus doses of 0.002 mg/kg hydromorphone permitted every 8 min. In case of PCIA analgesic inadequate (VAS ≥ 40), dynastat 40 mg would be administered as an alternative rescue modality. Tropisetron 5 mg or palonosetron 0.25 mg could be administered to combat postoperative nausea and vomiting.

### Data collection and follow-up

During the preoperative interview, demographic characteristics, educational background, work type, and history of cigarette smoking and alcohol consumption were recorded. Besides, the general sleep quality within 1 month was recorded by a scale with three levels (poor, fair, and good). At the same time, patients were instructed on how to use the VAS to describe the pain they were experiencing, and how to use the PCIA device to control the pain when necessary. After surgery, the intraoperative parameters including surgery type and duration, anesthesia duration, lymphadenectomy, adhesion loosening, and pathologic diagnosis were also recorded.

During the follow-up period, VAS at rest and during coughing was recorded on the first morning (8:00 a.m.) after surgery. In the meantime, the use of rescue analgesia, postoperative sleep quality, and the degree of satisfaction (bad, fair, good, and excellent) to the pain management were recorded.

### End-points

The primary outcome was the occurrence of postoperative inadequate analgesia. Once patient experience at least one of the following situations during the first night and morning after surgery: require extra analgesic drug; report moderate-to-severe pain (VAS ≥ 4) at rest; report poor sleep quality; report bad satisfaction with pain control, they were defined as postoperative inadequate analgesia.

### Genotype analysis

Blood samples were collected in tubes containing ethylenediaminetetraacetic acid 1 h after CVC was implemented and were then stored at − 80 °C. Genomic DNA was extracted from whole blood for genetic analysis by using Blood Genomic DNA Mini Kit (Biomed Corporation, China) according to the manufacturer’s recommendations. DNA samples were then stored at − 20 °C. SNPs were genotyped using a KASP™ genotyping assay (Rui Biotechnology, Beijing, China) as previously described [30, 31].

Quality control was performed to ensure the robust genetic association: SNPs with call rates of < 95%, Minor Allele Frequency (MAF) <  0.05, or Hardy-Weinberg equilibrium (HWE) of *p* <  0.05 were excluded. Linkage disequilibrium (LD) was calculated from the patients’ genotypes. When strong LD (r^2^ > 0.9) was present in one gene, we only included one SNP from each pairs of SNP in the association study. Finally, there were 28 SNPs among the 18 candidate genes passed all quality control filters. (See Table 1).

**Table 1:** Description of all single nucleotide polymorphisms analyzed

Gene	Polymorphism	Functional Consequence	Variant	Major/minor allele frequency	Hardy Weinberg *p*-value
*ABCB1*	rs1045642	Synonymous codon	A > G	0.62/0.38	0.37
	rs1128503	Synonymous codon	A > G	0.67/0.33	0.63
*ADRB1*	rs1801252	Missense	A > G	0.83/0.17	0.46
	rs1801253	Missense	G > C	0.74/0.26	1
*CACNA1E*	rs3845446	Intron variant	T > C	0.7/0.3	0.61
*COMT*	rs4633	Synonymous codon	C > T	0.72/0.28	0.86
	rs4680	Missense	G > A	0.72/0.28	1
*DRD2*	rs6277	Synonymous codon	G > A	0.94/0.06	0.55
*ESR1*	rs9340799	Intron variant	A > G	0.81/0.19	0.25
*KCNJ6*	rs6517442	Upstream variant	C > T	0.73/0.27	0.07
	rs2070995	synonymous codon	T > C	0.61/0.39	0.55
*OPRM1*	rs1799971	Intron variant	A > G	0.69/0.31	0.50
	rs677830	Intron variant	C > T	0.89/0.11	0.46
	rs540825	Intron variant	A > T	0.92/0.08	1
*P2RX7*	rs7958311	Intron variant	G > A	0.52/0.48	0.67
*P2RY12*	rs3732765	Intron variant	G > A	0.87/0.13	1
*SCN11A*	rs33985936	Missense	C > T	0.89/0.11	0.48
	rs11709492	Intron variant	C > T	0.74/0.26	0.71
*SCN10A*	rs6795970	Missense	A > G	0.86/0.14	0.38
*SCN9A*	rs6746030	Intron variant	A > G	0.95/0.05	0.36
	rs4286289	Intron variant	C > A	0.56/0.44	1
	3312G > T	Missense	G > T	0.9/0.1	0.69
*TAOK3*	rs795484	Intron variant	T > C	0.68/0.32	0.74
	rs1277441	Intron variant	G > A	0.59/0.41	0.14
*TGFB1*	rs1800469	Downstream variant	A > G	0.51/0.49	0.26
*TRPV1*	rs8065080	Missense	T > C	0.64/0.36	0.22
*UGT2B7*	rs7439366	Missense	T > C	0.69/0.31	0.87
*CREB1*	rs2952768	None	T > C	0.57/0.43	0.77

Abbreviations: *ABCB1* ATP binding cassette subfamily B member 1, *ADRB1* adrenoceptor beta 1, *CACNA1E* calcium voltage-gated channel subunit alpha1 E, *COMT* catechol-O-methyltransferase, CREB1 cAMP responsive element binding protein 1, *DRD2* dopamine receptor D2, *ESR1* estrogen receptor 1, *KCNJ6* potassium voltage-gated channel subfamily J member 6; *OPRM1* opioid receptor mu 1, *P2RX7* purinergic receptor P2X 7; *P2RY12* purinergic receptor P2Y12, *SCN11A* sodium voltage-gated channel alpha subunit 11; *SCN10A* sodium voltage-gated channel alpha subunit 10, *SCN9A* sodium voltage-gated channel alpha subunit 9, *TAOK3* TAO kinase 3, *TGFB1* transforming growth factor beta 1, *TRPV1* transient receptor potential cation channel subfamily V member 1, *UGT2B7* UDP glucuronosyltransferase family 2 member B7

### Statistical analysis

Statistical analysis was completed with the SPSS 24.0 (SPSS Inc., Chicago, IL). Continuous variables were expressed as means and standard deviations (SDs) or as medians and interquartile range, and categorical variables as counts and percentages. Differences between two groups were evaluated by Student’s t-test or the Mann-Whitney test for continuous variables, and Chi-squared test or Fisher’s exact test for categorical variables. For analyzing the association between SNPs and inadequate analgesia, odds ratios (ORs) and 95% confidence intervals (CI) were calculated by logistic regression analysis adjusted for potential risk factors. Four genetic models (co-dominant, dominant, recessive and overdominant) were evaluated for association of polymorphisms with risk of inadequate analgesia. HWE was assessed by SNPStats software [32]. The linkage disequilibrium and pairwise LD coefficients were implemented with Haploview 4.2 (Daly Lab: Cambridge, MA, USA, 2008). *P* value < 0.05 was considered significant.

Power analysis was done using QUANTO (University of Southern California, Los Angeles, CA). For the analyses of associations with postoperative inadequate analgesia, with the sample size of 198 and a modest Type I error rate of 5%, the analysis had more than 90% power to detect an OR of 2.15 for SNPs with an MAF ≥ 0.11 under dominant model and more than 99% power to detect an OR of 0.41 for SNPs with an MAF ≥ 0.26 under recessive model.

## Results

### Patient characteristics

**Fig. 1:**
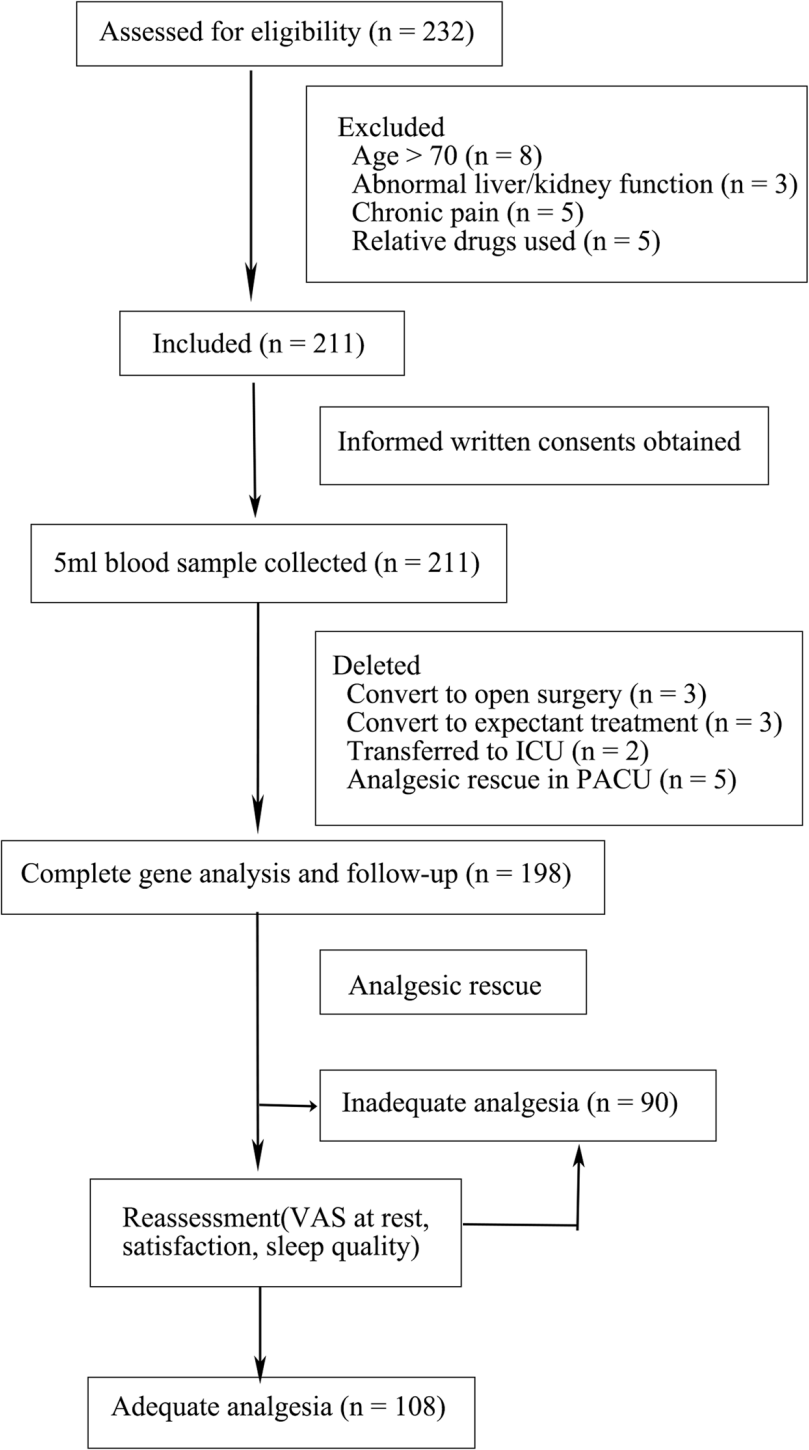
Flowchart for inclusion and follow-up in the study. Abbreviations: ICU = intensive care unit; PACU = postanesthesia care unit; VAS = visual analog scale

From July 2018 to January 2019, a total of 232 patients underwent single-port VATS at our center. Two hundred eleven patients met the inclusion criteria voluntarily participate in this study. Thirteen patients were withdrawn due to conversion to open surgery (*n* = 3) or expectant treatment (n = 3), transferred to the intensive care unit after operation (*n* = 2), and received rescue analgesics in PACU (*n* = 5). Therefore, the data from 198 patients entered the final analysis as shown in Fig. 1.

The overall demographic and clinical characteristics are shown in Table 2 and Table 3. All enrolled patients were Chinese population. The baseline cohort comprised 115 females (58.1%) and 83 males (41.9%), aged 37 to 70 years (mean 58.0 years). Postoperative inadequate analgesia was observed in 90 patients (90/198, 45.5%), including 66 patients with moderate-to-severe pain at rest (VAS ≥ 4), 45 patients required extra analgesic drug, 68 patients had bad sleep quality, and 30 patients were unsatisfactory to the pain management. Except for age and education level, there were no significant differences regarding demographic variables and clinical characteristics between patients with and without inadequate analgesia. The patients with inadequate analgesia were significant younger, and had higher education level than patients without inadequate analgesia. These variables were included as covariates in the regression model of genetic association analysis.

**Table 2:** Distribution of socio-demographic characteristics between patients with and without postoperative inadequate analgesia

Variables	Adequate analgesia (*n* = 108)	Inadequate analgesia (*n* = 90)	*P*-Value
Age (y) (mean ± SD)	60.08 ± 6.79	55.42 ± 9.03	< 0.001
Sex			0.11
Female	70 (63.1%)	45 (51.7%)	
Male	41 (36.9%)	42 (48.3%)	
BMI (mean ± SD)	22.86 ± 2.84	23.10 ± 2.57	0.54
Weight (mean ± SD)	60.92 ± 9.34	63.31 ± 9.30	0.07
History of Cigarette Smoking			0.62
Yes	22 (20.4%)	21 (24.1%)	
No	86 (79.6%)	69 (75.9%)	
History of Alcohol Consumption			1.00
Yes	24 (22.2%)	10 (22.2%)	
No	84 (77.8%)	70 (77.8%)	
Educational Level			< 0.001
Low	52 (48.1%)	23 (25.6%)	
Medium	46 (42.6%)	35 (38.9%)	
High	10 (9.3%)^*^	32 (35.6%)	
Exercise			0.26
Yes	49 (45.4%)	48 (53.3%)	
No	59 (54.6%)	42 (46.7%)	
Preoperative Sleep Quality			0.33
Poor	18 (16.7%)	21 (23.3%)	
Fair	43 (39.8%)	28 (31.1%)	
Good	47 (43.5%)	41 (45.6%)	
Major Surgery within 2 Years			0.51
Yes	9 (8.3%)	10 (11.1%)	
No	99 (91.7%)	80 (88.9%)	

Abbreviations: BMI = body mass index; SD = standard deviation

**Table 3:** Distribution of clinical characteristics between patients with and without postoperative inadequate analgesia

Variables	Adequate analgesia (*n* = 108)	Inadequate analgesia (*n* = 90)	*P*-Value
Surgery time (median [IQR])	82.5 (60–105)	85 (65–100)	0.97
Anesthesia time (median [IQR])	110 (83.5–135)	110 (91.5–128.5)	0.89
Surgery end-time (median [IQR]	15.5 (13.3–18)	16 (14–18.6)	0.27
Section Parts			0.70
One part	90 (83.3%)	76 (84.4%)	
Two parts	15 (13.9%)	13 (14.4%)	
Three parts	3 (2.8%)	1 (1.1%)	
Surgery Type			0.49
Wedge resection	17 (15.7%)	8 (8.9%)	
Segmentectomy	21 (19.4%)	16 (17.8%)	
Lobectomy	69 (63.9%)	65 (72.2%)	
Pneumonectomy	1 (0.9%)	1 (1.1%)	
Lymph Node Dissection			0.35
Yes	91 (84.3%)	80 (88.9%)	
No	17 (15.7%)	10 (11.1%)	
Adhesion Loosening			0.28
Yes	54 (50.0%)	38 (42.2%)	
No	54 (50.0%)	52 (57.8%)	
Pathologic Diagnosis			0.47
Low-Grade	9 (8.3%)	12 (13.3%)	
High-Grade	73 (67.6%)	60 (66.7%)	
Benign	26 (24.1%)	18 (20.0%)	

Abbreviations: *IQR* interquartile range

### Association analysis

The genotyping call rate was 100%. All the selected SNPs met the HWE criterion (*p* <  0.05) and without low MAF (*p* <  0.05). Strong LDs in *SCN11A* (Additional file 1: Figure S1) were identified in our sample, and we included only one SNP from each pair of SNP in the association study. Thus, 28 SNPs among 18 genes were assessed for further association analysis.

The distribution of the allele and genotype frequencies of the remaining 28 SNPs in patients with and without inadequate analgesia is summarized in Additional file 2: Table S1. Significant associations between genetic mutations and postoperative inadequate analgesia were detected in six SNPs among five genes (*ESR1*, *P2RY12*, *SCN11A*, *SCN9A*, and *TAOK3*) by the logistic regression (see Table 4). After adjusting for potential confounders, four SNPs remained significant: rs33985936 (*SCN11A*), rs11709492 (*SCN11A*), rs6795970 (*SCN10A*), and 3312G > T (*SCN9A*).

**Table 4:** Logistic regression analyses of associations between SNPs and risk of postoperative inadequate analgesia

Gene	SNP	Model	Genotype	Adequate analgesia	Inadequate analgesia	OR (95% CI) unadjusted	*P*-Value unadjusted	OR (95% CI) adjusted	*P*-Value adjusted
*ESR1*	rs9340799	Recessive	A/A-G/A	99 (91.7%)	89 (98.9%)	1.00	0.01	1.00	0.020
			G/G	9 (8.3%)	1 (1.1%)	0.12 (0.02–0.99)		0.13 (0.02–1.08)	
*P2RY12*	rs3732765	Dominant	G/G	76 (70.4%)	75 (83.3%)	1.00	0.031	1.00	0.180
			G/A-A/A	32 (29.6%)	15 (16.7%)	0.48 (0.24–0.95)		0.61 (0.29–1.28)	
*SCN11A*	rs33985936	Dominant	C/C	89 (82.4%)	66 (73.3%)	1.00	0.12	1.00	0.042
			T/C-T/T	19 (17.6%)	24 (26.7%)	1.70 (0.86–3.37)		2.15 (1.02–4.52)	
	rs11709492	Dominant	C/C	51 (47.2%)	56 (62.2%)	1.00	0.03	1.00	0.005
			T/C-T/T	57 (52.8%)	34 (37.8%)	0.54 (0.31–0.96)		0.41 (0.22–0.77)	
*SCN10A*	rs6795970	Dominant	G/G	84 (77.8%)	59 (65.6%)	1.00	0.06	1.00	0.026
			G/A-A/A	24 (22.2%)	31 (34.4%)	1.84 (0.98–3.45)		2.14 (1.09–4.21)	
*SCN9A*	rs6746030	Dominant	G/G	94 (87%)	86 (95.6%)	1.00	0.032	1.00	0.067
			G/A-A/A	14 (13%)	4 (4.4%)	0.31 (0.10–0.99)		0.35 (0.10–1.16)	
	3312G > T	Dominant	G/G	96 (88.9%)	66 (73.3%)	1.00	0.005	1.00	0.011
			T/G-T/T	12 (11.1%)	24 (26.7%)	2.91 (1.36–6.22)		2.85 (1.25–6.51)	
*TAOK3*	rs1277441	Dominant	T/T	33 (30.6%)	40 (44.4%)	1.00	0.044	1.00	0.13
			T/C-C/C	75 (69.4%)	50 (55.6%)	0.55 (0.31–0.99)		0.61 (0.32–1.15)	

Abbreviations: *CI* confidence intervals, *ESR1* estrogen receptor 1, *OR* odds ratios, *P2RY12* purinergic receptor P2Y12, *SCN11A* sodium voltage-gated channel alpha subunit 11, *SCN10A* sodium voltage-gated channel alpha subunit 10, *SCN9A* sodium voltage-gated channel alpha subunit 9, *SNP* single nucleotide polymorphism, *TAOK3* TAO kinase 3

For *SCN11A*, two SNPs (i.e., rs33985936, rs11709492) were associated with the occurrence of inadequate analgesia. For rs33985936, individuals who carried the rare T allele (TC + TT vs. CC) had a 2.15-fold increase in the odds of reporting inadequate analgesia. The rare T allele carriers of rs11709492 were found to be associated with decreased risk of inadequate analgesia (OR = 0.41, 95% CI: 0.22–0.77, *p* = 0.005).

For *SCN10A* rs6795970, patients with GA/ AA genotype had a 2.14-fold increase in the odds of reporting inadequate analgesia compared to GG genotype.

For *SCN9A* 3312G > T, patients who were heterozygous or homozygous for the rare T allele (TG + TT vs. GG) had a 2.85-fold increase in the odds of reporting postoperative inadequate analgesia.

## Discussion

In the present study, the incidence of postoperative inadequate analgesia in patients undergoing single-port VATS was 45.5%. Twenty-eight SNPs among 18 genes involved in pain perception and modulation were selected to test the association between genetic polymorphisms and postoperative inadequate analgesia. After adjusting for confounding factors, significant association with inadequate analgesia was found in four SNPs (rs11709492, rs33985936, rs6795970, and 3312G > T) of genes encoding voltage-gated sodium channels.

Although numerous measures have been developed for the management of postoperative pain, the proportion of patient experience moderate to severe postoperative pain after thoracotomy was relatively high [2]. In the present study, the inadequate analgesia was happened in 45.5% of patients who received intercostal nerve block and standard PCIA with hydromorphone after single-port VATS. This result was consistent with previous reports [3, 33]. Considering some patients were unwilling to take any extra analgesic drug even though they were unsatisfactory to the analgesia effect or their sleep was disturbed by pain, we defined postoperative inadequate analgesia as not only patients with moderate to severe pain and required extra analgesic drug, but also patients with bad sleep quality and low satisfaction. This definition comprehensively comprises the real patients with inadequate analgesia.

Our results indicated that four SNPs in genes encoding voltage-gated sodium channels (VGSCs) were associated with pain perception after sing-port VATS. VGSCs play a key role in the initiation and transmission of action potentials in excitable cells [34]. More than 1000 disease-related mutations have been discovered in nine VGSC-encoding genes [35]. It has been widely recognized that the changes of VGSC expression are involved in the sensitization of sensory neurons in many acute and chronic pain conditions [36].

*SCN11A* encodes one member of the sodium channel alpha subunit gene family NaV1.9, and is highly expressed in nociceptive neurons of dorsal root ganglia and trigeminal ganglia. Mutations in this gene have been associated with hereditary pain syndromes [37]. In this study, we identified two SNPs (rs33985936, rs11709492) were associated with inadequate analgesia. The mutation of rs33985936 (2725C > T) causes amino acid substitution Val909Ile which leads to the changes in intermolecular force of Nav1.9 [17]. Previous study reported that subjects who carrying the minor allele of rs33985936 were more sensitive to pain, while patients carrying the minor allele of rs11709492 have lower pain sensitivity [17]. This was consistent with our results that individuals who carried the rare T allele of rs33985936 had a 2.3-fold increase in the odds of reporting inadequate analgesia, and the rare T allele of rs11709492 were found to be associated with decreased risk of inadequate analgesia.

*SCN10A* and *SCN9A* encode Nav1.8 and Nav1.7 sodium channels, respectively. They are preferentially expressed in dorsal root ganglion sensory neurons and sympathetic ganglia and significantly influence nociceptor excitability [15, 38, 39]. Recent genetic studies have identified rare and common mutations in *SCN9A* and *SCN10A* as contributory both in chronic pain conditions and postoperative pain [15, 16, 39]. Guangyou Duan et al. reported that 3312G > T (*SCN9A*), a nonsynonymous SNP leading to the amino acid substitution V1104 L in human Nav1.7, was associated with postoperative inadequate analgesia [16]. Patients carrying the 3312G allele had a higher incidence of inadequate analgesia than those carrying the 3312 T allele. They also demonstrated an association between *SCN10A* rs6795970 and higher thresholds for mechanical pain in experimental pain testing [15]. However, in our study, we identified that patients with the 3312Tallele (3312G > T) and A allele (rs6795970) had a higher risk of presenting with inadequate analgesia.

As mentioned before, *OPRM1*and *COMT* play an important role in endogenous pain modulation and opioid analgesia. De Gregori M et al. found that genetic polymorphisms in *OPRM1* (rs540825), *COMT* (rs4680) and *ESR1* (rs9340799) have clinical effect on the morphine consumption and pain scores after major surgery [40]. The rs1799971 polymorphisms of the *OPRM1* gene is related to the analgesic effect and sufentanil consumption in Chinese Han patients after radical operation of lung cancer [9]. Nevertheless the correlations were not confirmed in our present study. For *ESR1* (rs9340799), the frequencies of G allele in patients with postoperative inadequate analgesia was less than in patients without (15% vs. 23%). Compared with the AA and AG genotypes, the homozygous genotype GG appeared to decrease the risk for postoperative inadequate analgesia (OR = 0.12, 95% CI: 0.02–0.99, *p* = 0.01). After adjusting for potential confounders, although the *P*-value of the correlation was statistically significant (*p* < 0.05), the odds ratio didn’t have clinical significance (OR = 0.13, 95% CI: 0.02–1.08). So we didn’t include the rs9340799 (*ESR1*) to the final results.

This inconsistence may partly due to the different subjects and different surgery type. The general situation of the patients included in the present study differed from previous studies, and it is known that factors such as age, race, and gender all may affect the patient’s pain sensitivity [41, 42]. Then, the present study focused on patients undergoing single-port thoracoscopic surgery. The mechanisms (inflammatory pain, mechanical traction pain, and intercostal neuralgia) and degrees of postoperative pain may vary from the type of surgery. This difference may cause the same polymorphism to show inconsistent or even completely opposite results in different surgeries [20, 43]. Besides, the findings identified by Guangyou Duan et al. were based on experimental pain tests performed on healthy volunteers [16]. This suggests that we should be rigorous to use these results in assessing pain sensitivity of patients after surgery. Therefore, further replication studies are warranted to confirm these findings.

There are some limitations in the present study. First, the postoperative inadequate analgesia was assessed within the first 24 h after surgery. In the previous study, we found that the intense pain after single-port VATS is sustained within 24 h especially during the first night and the first morning after surgery and majority of patients achieved satisfactory pain relief after 24 h [29]. So we only followed up until the first night and first morning after surgery. Second, these findings are specific to the patients underwent single-port VATS. The associations found in this study may differ in thoracotomy. Finally, due to the exploration nature of the study, the sample size was relative small and we did not replicate the study in a validation cohort. Future studies with a larger sample size may increase the power to detect differences in other candidate genes.

## Conclusions

In summary, our findings suggest that polymorphisms in voltage-gated sodium channels genes play a role in the inadequate postoperative analgesia after single-port VATS. The genes and SNPs found in this study may help to identify, prevent, and targetedly treat patients with high risk of experiencing inadequate postoperative pain. This, in turn, may improve the postoperative rehabilitation and reduce short- and long-term morbidity. Future studies are warranted to confirm our findings and to determine if these associations are present in the opioid consumption analysis.

## Supplementary information

**Additional file 1: Figure S1**. LD plots of SNPs of the *SCN11A* gene. Identifies the linkage disequilibrium of SNPs among *SCN11A* gene.

**Additional file 2: Table S1**. Genotype and allele distributions of polymorphisms in patients with and without postoperative inadequate analgesia.

## Data Availability

All relevant data and materials are presented in the manuscript.

## Abbreviations

ABCB1: ATP binding cassette subfamily B member 1
ADRB1: Adrenoceptor beta 1
CACNA1E: Calcium voltage-gated channel subunit alpha1 E
CI: Confidence intervals
COMT: Catechol-O-methyltransferase
CREB1: CAMP responsive element binding protein 1
CVC: Central venous catheterization
DRD2: Dopamine receptor D2
ESR1: Estrogen receptor 1
HWE: Hardy-Weinberg equilibrium
KCNJ6: Potassium voltage-gated channel subfamily J member 6
LD: Linkage disequilibrium
MAF: Minor allele frequency
OPRM1: Opioid receptor mu 1
ORs: Odds ratios
P2RX7: Purinergic receptor P2X 7
P2RY12: Purinergic receptor P2Y12
PACU: Postanesthesia care unit
PCIA: Patient-controlled intravenous
SCN10A: Sodium voltage-gated channel alpha subunit 10
SCN11A: Sodium voltage-gated channel alpha subunit 11
SCN9A: Sodium voltage-gated channel alpha subunit 9
SDs: Standard deviations
SNPs: Single nucleotide polymorphisms
TAOK3: TAO kinase 3
TGFB1: Transforming growth factor beta 1
TRPV1: Transient receptor potential cation channel subfamily V member 1
UGT2B7: UDP glucuronosyltransferase family 2 member B7
VAS: Visual analog scale
VATS: Video-assisted thoracoscopic surgery
VGSCs: Voltage-gated sodium channels
