# Supplementary material for: Single nucleotide polymorphisms associated with postoperative inadequate analgesia after single-port VATS in Chinese population
Source: BMC Anesthesiol. 2020 Feb 5;20:38. doi: 10.1186/s12871-020-0949-6 (PMC7003404; doi:10.1186/s12871-020-0949-6)
Supplement: Supplementary file 1 — Additional file 1: Figure S1. LD plots of SNPs of the SCN11A gene. Identifies the linkage disequilibrium of SNPs among SCN11A gene. [file 12871_2020_949_MOESM1_ESM.docx]

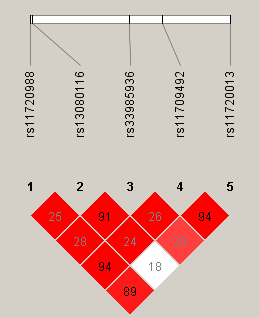


Figure S1. LD plots of SNPs of the *SCN11A* gene. The top horizontal bar illustrates the location of SNPs on a physical scale. Colors indicate the level of D’ (white = low D’, red = high D’), while numbers represent the r^2^ value (no number where r^2^ = 1). The rs11709492 SNP was genotyped as a proxy (r^2^ > 0.9) for rs11700988 and rs117020013. The rs33985936 SNP was genotyped as a proxy (r^2^ > 0.9) for rs13080116.
