# Supplementary material for: Single nucleotide polymorphisms associated with postoperative inadequate analgesia after single-port VATS in Chinese population
Source: BMC Anesthesiol. 2020 Feb 5;20:38. doi: 10.1186/s12871-020-0949-6 (PMC7003404; doi:10.1186/s12871-020-0949-6)
Supplement: Supplementary file 2 — Additional file 2: Table S1. Genotype and allele distributions of polymorphisms in patients with and without postoperative inadequate analgesia. [file 12871_2020_949_MOESM2_ESM.docx]

Table S1. Genotype and allele distributions of polymorphisms in patients with and without postoperative inadequate analgesia

| Gene | Polymorphism | Genotype/ allele | Adequate analgesia | Inadequate analgesia | P-Value |
| --- | --- | --- | --- | --- | --- |
| *ABCB1* | rs1045642 | C/C | 40 (0.37) | 32 (0.36) | 0.669 |
|  |  | C/T | 52 (0.48) | 48 (0.53) |  |
|  |  | T/T | 16 (.015) | 10 (0.1) |  |
|  |  | C | 132 (0.61) | 112 (0.62) | 0.821 |
|  |  | T | 84 (0.39) | 68 (0.38) |  |
|  | rs1128503 | C/C | 14 (0.13) | 5 (0.06) | 0.206 |
|  |  | T/C | 47 (0.44) | 44 (0.49) |  |
|  |  | T/T | 47 (0.44) | 41 (0.46) |  |
|  |  | T | 141 (0.65) | 126 (0.70) | 0.318 |
|  |  | C | 75 (0.35) | 54 (0.30) |  |
| *ADRB1* | rs1801252 | A/A | 72 (0.67) | 62 (0.69) | 0.699 |
|  |  | A/G | 33 (0.31) | 27 (0.3) |  |
|  |  | G/G | 3 (0.03) | 1 (0.01) |  |
|  |  | A | 177 (0.82) | 151 (0.84) | 0.609 |
|  |  | G | 39 (0.18) | 29 (0.16) |  |
|  | rs1801253 | C/C | 63 (0.58) | 47 (0.52) | 0.421 |
|  |  | C/G | 40 (0.37) | 35 (0.39) |  |
|  |  | G/G | 5 (0.05) | 8 (0.09) |  |
|  |  | C | 166 (0.77) | 129 (0.72) | 0.239 |
|  |  | G | 50 (0.23) | 51 (0.28) |  |
| *CACNA1E* | rs3845446 | A/A | 52 (0.48) | 47 (0.52) | 0.315 |
|  |  | A/G | 48 (0.44) | 32 (0.36) |  |
|  |  | G/G | 8 (0.07) | 11 (0.12) |  |
|  |  | A | 152 (0.70) | 126 (0.70) | 0.938 |
|  |  | G | 64 (0.30) | 54 (0.30) |  |
| *COMT* | rs4633 | C/C | 56 (0.52) | 45 (0.50) | 0.498 |
|  |  | C/T | 46 (0.43) | 36 (0.40) |  |
|  |  | T/T | 6 (0.06) | 9 (0.10) |  |
|  |  | C | 158 (0.73) | 126 (0.70) | 0.488 |
|  |  | T | 58 (0.27) | 54 (0.30) |  |
|  | rs4680 | C/C | 60 (0.56) | 44 (0.49) | 0.416 |
|  |  | C/T | 42 (0.39) | 37 (0.41) |  |
|  |  | T/T | 6 (0.06) | 9 (0.10) |  |
|  |  | C | 162 (0.75) | 125 (0.69) | 0.218 |
|  |  | T | 54 (0.25) | 55 (0.31) |  |
| *DRD2* | rs6277 | C/C | 92 (0.85) | 82 (0.91) | 0.352 |
|  |  | C/T | 15 (0.14) | 8 (0.09) |  |
|  |  | T/T | 1 (0.01) | 0 (0) |  |
|  |  | C | 199 (0.92) | 172 (0.96) | 0.163 |
|  |  | T | 17 (0.08) | 8 (0.04) |  |
| *ESR1* | rs9340799 | A/A | 68 (0.63) | 64 (0.71) | 0.062 |
|  |  | A/G | 31 (0.29) | 25 (0.28) |  |
|  |  | G/G | 9 (0.08) | 1 (0.01) |  |
|  |  | A | 167 (0.77) | 153 (0.85) | 0.053 |
|  |  | G | 49 (0.23) | 27 (0.15) |  |
| *KCNJ6* | rs6517442 | C/C | 5 (0.05) | 4 (0.04) | 0.591 |
|  |  | T/C | 52 (0.48) | 37 (0.41) |  |
|  |  | T/T | 51 (0.47) | 49 (0.54) |  |
|  |  | T | 154 (0.71) | 135 (0.75) | 0.409 |
|  |  | C | 62 (0.29) | 45 (0.25) |  |
|  | rs2070995 | A/A | 19 (0.18) | 13 (0.14) | 0.720 |
|  |  | G/A | 50 (0.46) | 40 (0.44) |  |
|  |  | G/G | 39 (0.36) | 37 (0.41) |  |
|  |  | G | 128 (0.59) | 114 (0.63) | 0.408 |
|  |  | A | 88 (0.41) | 66 (0.37) |  |
| *OPRM1* | rs1799971 | A/A | 48 (0.44) | 45 (0.50) | 0.373 |
|  |  | A/G | 53 (0.45) | 36 (0.40) |  |
|  |  | G/G | 7 (0.06) | 9 (0.10) |  |
|  |  | A | 149 (0.69) | 126 (0.70) | 0.827 |
|  |  | G | 67 (0.31) | 54 (0.30) |  |
|  | rs677830 | C/C | 82 (0.76) | 77 (0.86) | 0.115 |
|  |  | C/T | 23 (0.21) | 13 (0.14) |  |
|  |  | T/T | 3 (0.03) | 0 (0) |  |
|  |  | C | 187 (0.87) | 167 (0.93) | 0.046 |
|  |  | T | 29 (0.13) | 13 (0.07) |  |
|  | rs540825 | A/A | 88 (0.81) | 81 (0.90) | 0.197 |
|  |  | A/T | 19 (0.18) | 9 (0.10) |  |
|  |  | T/T | 1 (0.01) | 0 (0) |  |
|  |  | A | 195 (0.90) | 171 (0.95) | 0.077 |
|  |  | T | 21 (0.10) | 9 (0.05) |  |
| *P2RX7* | rs7958311 | A/A | 27 (0.25) | 27 (0.30) | 0.734 |
|  |  | A/G | 54 (0.50) | 42 (0.47) |  |
|  |  | G/G | 27 (0.25) | 21 (0.23) |  |
|  |  | A | 108 (0.50) | 96 (0.53) | 0.509 |
|  |  | G | 108 (0.50) | 84 (0.47) |  |
| *P2RY12* | rs3732765 | A/A | 3 (0.03) | 0 (0) | 0.053 |
|  |  | G/A | 29 (0.27) | 15 (0.17) |  |
|  |  | G/G | 76 (0.70) | 75 (0.83) |  |
|  |  | G | 181 (0.84) | 165 (0.92) | 0.019 |
|  |  | A | 35 (0.16) | 15 (0.08) |  |
| *SCN11A* | rs33985936 | C/C | 89 (0.82) | 66 (0.73) | 0.204 |
|  |  | C/T | 19 (0.18) | 23 (0.26) |  |
|  |  | T/T | 0 (0) | 1 (0.01) |  |
|  |  | C | 197 (0.91) | 155 (0.86) | 0.108 |
|  |  | T | 19 (0.09) | 25 (0.14) |  |
|  | rs11709492 | C/C | 51 (0.47) | 56 (0.62) | 0.034 |
|  |  | C/T | 47 (0.44) | 32 (0.36) |  |
|  |  | T/T | 10 (0.09) | 2 (0.02) |  |
|  |  | C | 149 (0.69) | 144 (0.80) | 0.013 |
|  |  | T | 67 (0.31) | 36 (0.20) |  |
| *SCN10A* | rs6795970 | A/A | 1 (0.01) | 1 (0.01) | 0.158 |
|  |  | G/A | 23 (0.21) | 30 (0.33) |  |
|  |  | G/G | 84 (0.78) | 59 (0.66) |  |
|  |  | G | 191 (0.88) | 148 (0.82) | 0.080 |
|  |  | A | 25 (0.12) | 32 (0.18) |  |
| *SCN9A* | rs6746030 | A/A | 1 (0.01) | 0 (0) | 0.104 |
|  |  | G/A | 13 (0.12) | 4 (0.04) |  |
|  |  | G/G | 94 (0.87) | 86 (0.96) |  |
|  |  | G | 201 (0.93) | 176 (0.98) | 0.029 |
|  |  | A | 15 (0.07) | 4 (0.02) |  |
|  | rs4286289 | G/G | 18 (0.17) | 20 (0.22) | 0.249 |
|  |  | T/G | 51 (0.47) | 47 (0.52) |  |
|  |  | T/T | 39 (0.36) | 23 (0.26) |  |
|  |  | T | 129 (0.60) | 93 (0.52) | 0.108 |
|  |  | G | 87 (0.40) | 87 (0.48) |  |
|  | 3312G>T | G/G | 96 (0.89) | 66 (0.73) | 0.011 |
|  |  | G/T | 12 (0.11) | 22 (0.24) |  |
|  |  | T/T | 0 (0) | 2 (0.02) |  |
|  |  | G | 204 (0.94) | 154 (0.86) | 0.003 |
|  |  | T | 12 (0.06) | 26 (0.14) |  |
| *TAOK3* | rs795484 | A/A | 14 (0.13) | 8 (0.09) | 0.421 |
|  |  | G/A | 48 (0.44) | 36 (0.40) |  |
|  |  | G/G | 46 (0.43) | 46 (0.51) |  |
|  |  | G | 140 (0.65) | 128 (0.71) | 0.182 |
|  |  | A | 76 (0.35) | 52 (0.29) |  |
|  | rs1277441 | C/C | 23 (0.21) | 16 (0.18) | 0.129 |
|  |  | T/C | 52 (0.48) | 34 (0.38) |  |
|  |  | T/T | 33 (0.31) | 40 (0.44) |  |
|  |  | T | 118 (0.55) | 114 (0.63) | 0.080 |
|  |  | C | 98 (0.45) | 66 (0.37) |  |
| *TGFB1* | rs1800469 | C/C | 27 (0.25) | 19 (0.21) | 0.709 |
|  |  | C/T | 59 (0.55) | 49 (0.54) |  |
|  |  | T/T | 22 (0.20) | 22 (0.24) |  |
|  |  | C | 113 (0.52) | 87 (0.48) | 0.429 |
|  |  | T | 103 (0.48) | 93 (0.52) |  |
| *TRPV1* | rs8065080 | C/C | 40 (0.37) | 36 (0.40) | 0.191 |
|  |  | C/T | 52 (0.48) | 48 (0.53) |  |
|  |  | T/T | 16 (0.15) | 6 (0.07) |  |
|  |  | C | 132 (0.61) | 120 (0.67) | 0.252 |
|  |  | T | 84 (0.39) | 60 (0.33) |  |
| *UGT2B7* | rs7439366 | A/A | 9 (0.08) | 9 (0.10) | 0.917 |
|  |  | G/A | 47 (0.44) | 39 (0.43) |  |
|  |  | G/G | 52 (0.48) | 42 (0.47) |  |
|  |  | G | 151 (0.70) | 123 (0.68) | 0.736 |
|  |  | A | 65 (0.30) | 57 (0.32) |  |
| *CREB1* | rs2952768 | A/A | 36 (0.33) | 27 (0.30) | 0.874 |
|  |  | A/G | 53 (0.49) | 47 (0.52) |  |
|  |  | G/G | 19 (0.18) | 16 (0.18) |  |
|  |  | A | 125 (0.58) | 101 (0.56) | 0.725 |
|  |  | G | 91 (0.42) | 79 (0.44) |  |

Abbreviations: ABCB1 = ATP binding cassette subfamily B member 1; ADRB1 = adrenoceptor beta 1; CACNA1E = calcium voltage-gated channel subunit alpha1 E; COMT = catechol-O-methyltransferase; CREB1 = cAMP responsive element binding protein 1; DRD2 = dopamine receptor D2; ESR1 = estrogen receptor 1; KCNJ6 = potassium voltage-gated channel subfamily J member 6; OPRM1 = opioid receptor mu 1; P2RX7 = purinergic receptor P2X 7; P2RY12 = purinergic receptor P2Y12; SCN11A = sodium voltage-gated channel alpha subunit 11; SCN10A = sodium voltage-gated channel alpha subunit 10; SCN9A = sodium voltage-gated channel alpha subunit 9; TAOK3 = TAO kinase 3; TGFB1 = transforming growth factor beta 1; TRPV1 = transient receptor potential cation channel subfamily V member 1; UGT2B7 = UDP glucuronosyltransferase family 2 member B7.
